# Filarioid nematodes in cattle, sheep and horses in Finland

**DOI:** 10.1186/1751-0147-50-20

**Published:** 2008-06-16

**Authors:** Milla Solismaa, Sauli Laaksonen, Minna Nylund, Elisa Pitkänen, Riitta Airakorpi, Antti Oksanen

**Affiliations:** 1 Finnish Food Safety Authority Evira, Fish and Wildlife Health Research Unit, PO Box 517, 90101 Oulu, Finland; 2 Finnish Food Safety Authority Evira, Inspecting veterinarian, Atria slaughterhouse, PO Box 147, 70101 Kuopio, Finland; 3 Finnish Food Safety Authority Evira, Inspecting veterinarian, Veljekset Rönkä Oy slaughterhouse, Mahlatie 7, 94200 Kemi, Finland

## Abstract

**Background:**

In autumn 2006, Finnish meat inspection data revealed lesions in tendons, muscles and ligaments of bovine hind legs leading to partial condemnation of carcasses. In gross pathological examination at Finnish Food Safety Authority Evira, Oulu (now Fish and Wildlife Health) Research Unit, *Onchocerca *sp. (Filarioidea; Onchocercidae) nematodes were detected in lesions. Due to this, a pilot study was made in order to find out what filarioid nematodes do occur in cattle, horses and sheep in Finland.

**Methods:**

Ventral skin biopsies from 209 dairy cattle and 42 horses, as well as blood samples from 209 cattle, 146 horses and 193 sheep, were collected from different parts of Finland and examined for microfilariae. Visceral organs and other tissues from 33 cattle with parasitic lesions were studied histopathologically.

**Results:**

*Onchocerca *sp. microfilariae (mf), 240 μm long, range 225–260 μm, 5.4 μm thick, were found in 37% of the skin biopsies of cattle. All blood samples from cattle, horses and sheep and skin biopsies from horses were negative for mf. Ventral skin microfilaria prevalence in cattle was higher in southern Finland than in the North (p = 0.001). Animal age and sampling time was not associated with mf prevalence. The infection was evenly distributed among young and older animals. Macroscopic lesions on tissues included greenish-grey discolouration and often oedema. In most of the lesions, small pale nodules were seen on the fasciae. Histopathologic examination of the samples revealed mild to intense infiltration with eosinophilic granulocytes and multifocal nodular lymphoplasmacytic aggregations were seen. In some samples, there were granulomatotic lesions with central necrotic tissue and cell detritus, surrounded by eosinophilic granulocytes, lympho-, plasma- and histiocytes and some multinucleated giant cells. Around living nematodes no or only weak inflammatory changes were observed.

**Conclusion:**

*Onchocerca *sp. infection in cattle was found to be common in Finland, but the amount of pathological changes leading to condemnation of infected parts is low compared to the mf prevalence. Pronounced pathological changes are distinct but rare and mild changes are difficult to distinguish. No other filarioid nematodes were observed from the animals and it appears that horses and sheep may be free from filarioid nematodes in Finland.

## Background

Filarioid nematodes are known to occur among domestic animals almost all over the world. The economically most important and also most abundant filarioid nematodes in cattle are *Setaria digitata, S. labiatopapillosa, S. marshalli, Onchocerca gibsoni, O. gutturosa, O. armillata, O. lienalis, O. ochengi, Parafilaria bovicola *and *Stephanofilaria *spp. Generally, species of *Onchocerca *are medium-sized filarioids which usually inhabit subcutaneous tissues, ligaments and aponeuroses of large mammals whereas species of *Setaria *are found in the abdominal cavities of artiodactyls. The filariids (*Parafilaria *and *Stephanofilaria*) are small to medium-sized subcutaneous parasites of certain mammals. All filarioid nematodes produce larvae (microfilariae, mf) into the skin (*Onchocerca *spp., *Parafilaria *spp. and *Stephanofilaria *spp.) or blood circulation (*Setaria *spp.) of the host where they are available to the haematophagous insects which operate as intermediate hosts and active vectors for the parasites [1].

In their normal definitive hosts, most species of filarioid nematodes are often very well adapted and they are well tolerated [2]. For example *Onchocerca *spp. of African cattle are generally believed to have a low pathogenicity [3]. Usually the damage caused by filarioid worms is the result of chronic inflammatory reactions around dead or dying worms or mf. Dead worms in the subcutaneous tissues usually become calcified and surrounded by dense fibrous tissue, causing little damage, but they may also act as a focus for bacteria and abscesses may develop in onchocercal nodules [2].

*Parafilaria bovicola *(Filarioidea; Filariidae) occurs in cattle mainly in Europe and Africa and causes cutaneous bleedings in live cattle and bruise-like lesions in the subcutaneous and intermuscular surfaces of affected carcasses [4]. In Sweden, *P. bovicola *was well established in the 1980's and since then has been responsible for substantial economic losses in beef production [5]. In Sweden it utilizes the face fly (*Musca autumnalis*) as a vector [6].

*Stephanofilaria *spp. are small filariid nematodes found in the subcutaneous tissues of bovids and cause sores and dermatitis in cattle in India, Malaysia and the U.S. [1]. The disease, stephanofilariosis is characterized by one or more circular or oblong areas of scaly, depilated, crusted skin at or near the umbilicus [7]. *Stephanofilaria *is transmitted by the horn fly *Haematobia irritans*. There are also indications of stephanofilarosis causing summer sore in cattle in Finland but the matter has not been thoroughly studied [8].*Setaria labiatopapillosa *is a common cosmopolitan parasite in the abdominal cavity of cattle, while *S. digitata *is found in Asian cattle. They are considered non-pathogenic [1,9]. However, immature stages of *S. digitata *invade the central nervous system of horses, sheep and goats causing lumbar paralysis and other CNS symptoms generally called cerebrospinal nematodosis [10].

Horses are commonly infected with *Setaria *spp. nematodes in Asia, Europe and America [11-13] and worldwide with *Onchocerca *spp. [14,15]. According to a report [16], *Onchocerca cervicalis *was fairly common among horses in Finland in the 1940's. *Onchocerca *infections of horses are most commonly seen clinically as a condition called fistulous withers [17].

*Parafilaria multipapillosa *is a parasite of the subcutaneous and inter-muscular connective tissues of horses in Eurasia, Africa and South America. Infection with the parasite results in condition known as "bloody sweat" or "summer bleeding" [1].

In North America a filarioid nematode, *Elaeophora schneideri*, lives in the arteries of domestic sheep [18]. Mule deer (*Odocoileus hemionus*) is believed to be the main definitive host of the worm which occurs also in other cervids [19]. Elaeophorosis caused by *Elaeophora elaphi *has also been found in the hepatic vessels of red deer (*Cervus elaphus*) in Europe [20]. In an aberrant host, like sheep, the skin dwelling microfilariae cause clinical sings in the form of dermatoses and pathological changes in retina and in the nasal and oral mucosa. The adult worms, on the other hand, damage the arteries where they live in [21]. Similar arterial damage is found in cattle associated with *Onchocerca armillata *and *Elaeophora *spp. infections in Tanzania [22].

Filarioid nematodes and their impacts on wild and semi-domesticated cervid ruminants have been under intense interest in Finland during the past few years. Attention to these nematodes was drawn in 2003 when there was an outbreak of peritonitis in reindeer caused by the nematode *Setaria tundra *(Filarioidea; Onchocercidae), the definitive host of which is assumed to be roe deer (*Capreolus capreolus*) [23]. The outbreak lead to economical losses to reindeer herders and it impaired meat hygiene. Recently, a new yet unidentified species of filarioid nematode was found in the lymphatic vessels of cervids (Laaksonen et al., unpublished). This new species is abundant especially among reindeer.

All filarioid nematodes are transmitted by haematophagous vectors. In temperate zones the transmission occurs in summer when the vectors are active [24]. Recent studies (Laaksonen et al., unpublished) give rise to the hypothesis that the currently high prevalence of filarioid nematodes in some animals in Finland may be associated with the ongoing climate change.

The detrimental effects of these filarioid outbreaks to the health and well-being of cervids, as well as to meat hygiene, raised questions about the impacts of filarioid nematodes on other meat production animals (cattle, sheep and horses). Studies on this area are scarce in Finland. Recent meat inspection data in autumn 2006 from Kuopio slaughterhouse revealed parasitic lesions in cattle and in examination at Finnish Food Safety Authority Evira, Oulu (currently Fish and Wildlife Health) Research Unit, *Onchocerca *sp. nematodes were found. Filarioid nematodes could posses a threat against meat producing animals and inflict economical losses to the meat and dairy industries. Therefore, elucidation of the filarioid situation in Finland was considered necessary.

The main aim of the study was to find out the species of filarioid nematodes occurring in cattle, sheep and horses in Finland, and their prevalence. The intention was to determine if the species causing pathological changes in slaughter cattle are the same as infecting cervids, and to describe infection dynamics.

## Methods

Material from cattle, sheep and horses was collected between 28 February and 24 September, 2007. Blood and skin samples from the animals were collected from slaughterhouses at Kuopio (Atria) (28.2.-14.6.07, 150 cattle skin samples of 17734 slaughtered), Kemi (Veljekset Rönkä Oy) (17.4.-22.5.07, 59 cattle skin samples of 673 slaughtered, 15.5.-24.9. 07, 193 sheep blood samples of 1104 slaughtered and 13 horse blood and skin samples) and Hautjärvi (Hannu Vainio Oy) (29 horse skin samples). In addition, blood samples from horses (133) were collected at different horse clinics by practising veterinarians in Oulu (17), Hyvinkää (21), Ypäjä (18), Lahti (19), Laukaa (20), Ylivieska (18) and Tampere (20). Blood samples from horses were mostly from half-breed trotters or mounts (riding-horses) visiting clinics for some undefined reason. Cattle and horse samples originated from all over Finland and sheep from the provinces of Lapland and Oulu. In slaughterhouses, samples were taken from all slaughtered horses during collecting period and cattle and sheep samples were collected randomly when labour was available. All the animals included in this study were over one year old and had been grazing outdoors in the previous summer and thus had been exposed to the potential vectors of filarioid nematodes. Blood samples were taken from all of the animals. Skin biopsies were taken from 209 cattle and from 42 horses. Tissue samples from 33 cattle (not included in the blood and skin monitoring) with lesions considered parasitic (subcutaneous and subfascial oedema and granulomas with greenish or yellowish coloration indicating eosinophilic infiltration, fibrotic or granulomatous fibrin depositions on visceral organs, especially on liver) were collected by the meat inspecting veterinarian during routine meat inspection from Kuopio slaughterhouse from 8 November, 2006, to 23 May, 2007. Tissues were delivered fresh to Evira, where they were dissected under stereo microscope for adult parasites, fixed in 10% neutral buffered formalin and routinely processed; embedded in paraffin, cut in 4 μm sections and stained with haematoxylin and eosin, and examined histopathologically. Samples included muscles and fasciae, tendons or ligaments of legs, flank or brisket from 24, liver samples from ten, lung samples from four and a spleen sample from one animal.

Blood samples were taken in 10 ml tubes (Venoject II, EDTA (K2): 19.5 mg, Terumo Europe N.V., Belgium). In laboratory, the examination for blood microfilariae was done by modified Knott's technique as described elsewhere [23].

Altogether 209 skin samples were collected from cattle (ages between 14 to 143 months). Skin samples were taken at the umbilical area following the examples given in literature [3,9]. In the beginning of the sample collection, also ear skin biopsies were taken from the first 60 animals but the procedure was discarded due to obviously lower sensitivity. Skin biopsies approximately 1 cm^2 ^in size were washed in tap water and cut into ten pieces with scissors. The pieces were incubated in fresh physiological saline for 24 h at room temperature (21–25°C). The tissues were discarded and microfilariae were concentrated by centrifugation for 12 minutes at 1600 g. The microfilariae were stained with 1% methylene blue and measured (n = 20).

Statistical analyses were made with Stata 9 (StataCorp, LP, USA) software. Finland was divided into two parts, North (Provinces of Lapland and Oulu) and South, in order to examine the spatial distribution of mf prevalence. The effect of age (group 1; <48 months, group 2; ≥48 months) to mf prevalence was evaluated using chi square tests. The level of significance was set at 5% (p < 0.05).

## Results

All blood samples examined were negative (cattle 209, horses 147, sheep 193). Also skin biopsies from the 42 horses examined were found negative for mf. Of the 209 cattle skin biopsies from umbilical area, 78 (37%) were positive for *Onchocerca *sp. mf (Table 1) and of the 60 samples collected from ears, 5% were positive.

**Figure 1:**
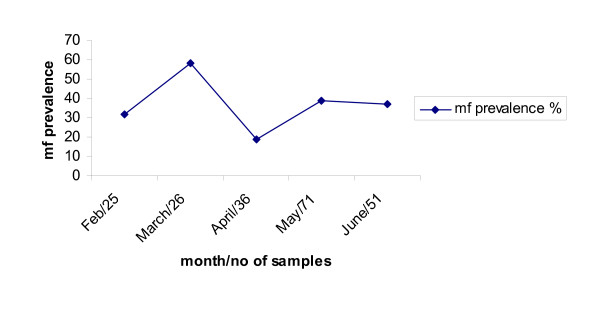
Temporal mf prevalence in slaughter cattle.

In the south the infection was more prevalent (p < 0.001). Neither age (p = 0.611) nor sampling time (Fig. 1) did affect the mf prevalence.

**Table 1:** Mf prevalence in skin samples of cattle in South and North (p < 0.001).

Area	Individuals	mf positive	%
1. South	109	54	49.5
2. North	100	24	24.0

Total	209	78	37.3

**Figure 2:**
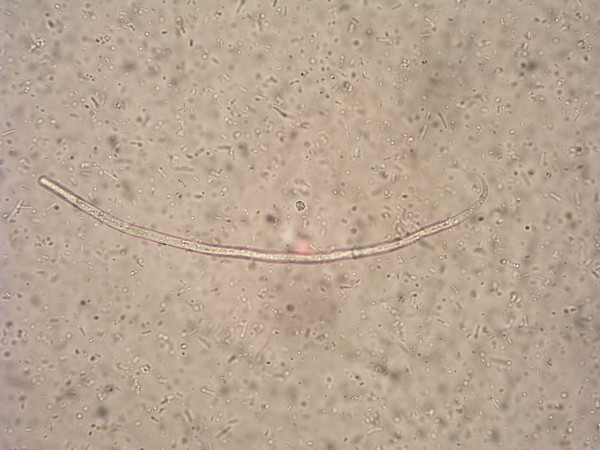
***Onchocerca*****sp. microfilaria found in bovine skin sample.**

Microfilariae from umbilical area and from ears were similiar and were identified as *Onchocerca *sp. by morphological features: Long and slender form with attenuated, pointed and twisted tail [1]. Unsheathed microfilariae were approximately 240 (SD 10.7) μm long (range 225–260 μm) and 5.4 μm thick (Fig. 2).

**Figure 3:**
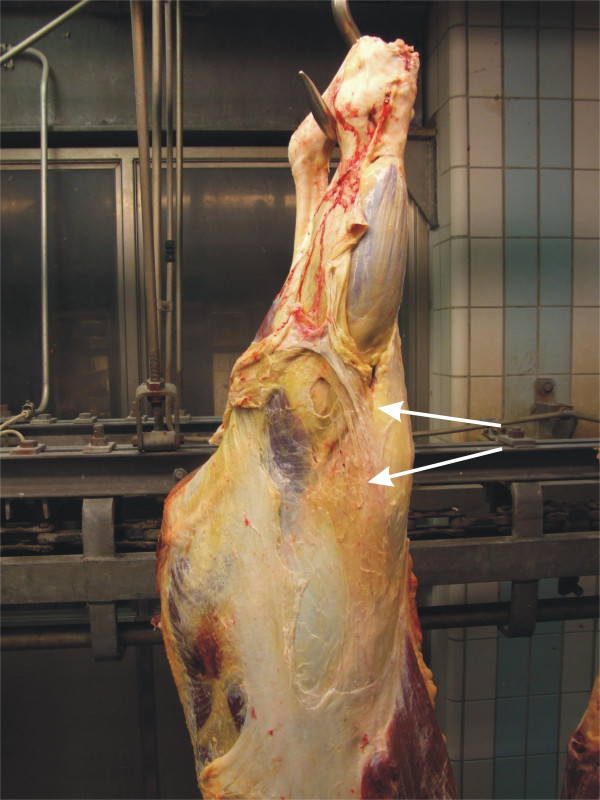
**Pathological changes in cattle.** The common changes (arrows) including oedema and greenish to grey colouration were seen on the flanks and hind legs.

**Figure 4:**
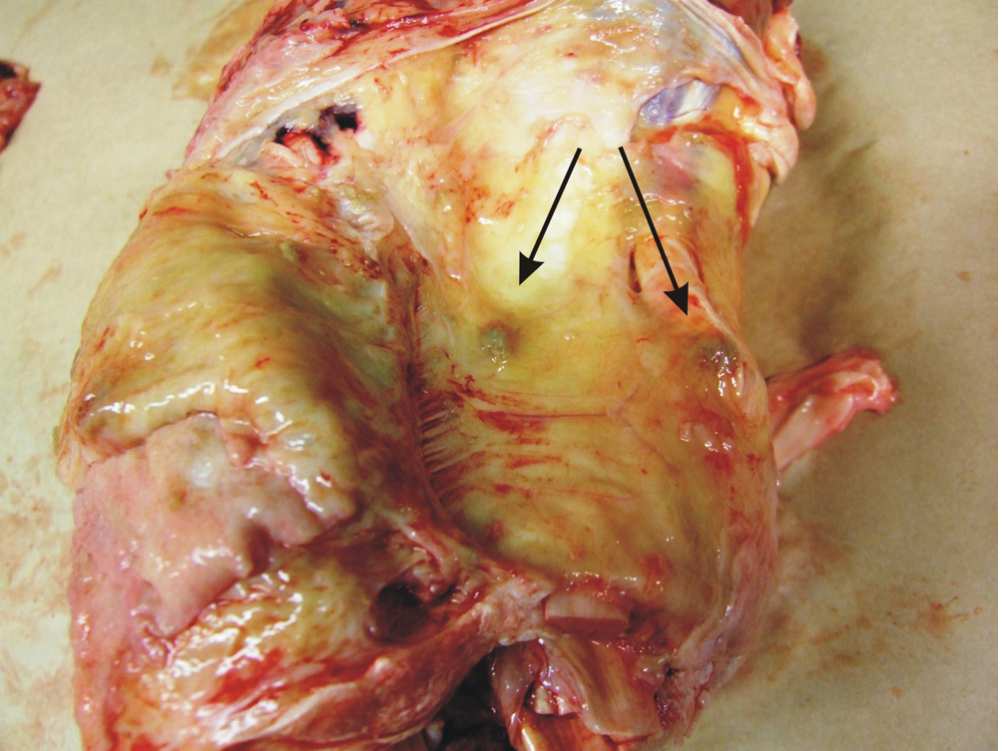
**Pathological changes in cattle.** Small pale nodules (arrows) were seen on the fasciae.

From the 33 cattle samples submitted to pathologic examination, macroscopic lesions on muscle fasciae and connective tissues included greenish-grey colouration and often oedema of the tissues (Fig. 3). In most of the samples, small (3–10 mm) pale granulomatous nodules were seen on the fasciae (Fig. 4). In liver samples there were multifocal small (2–6 mm), pale or yellowish nodules, most of them arranged in clusters. In lung samples only a few, tiny (2–3 mm) greenish coloured nodules were seen. On the surface of spleen some tiny, pale nodules and one larger nodule, about 5 mm in diameter were seen.

**Figure 5:**
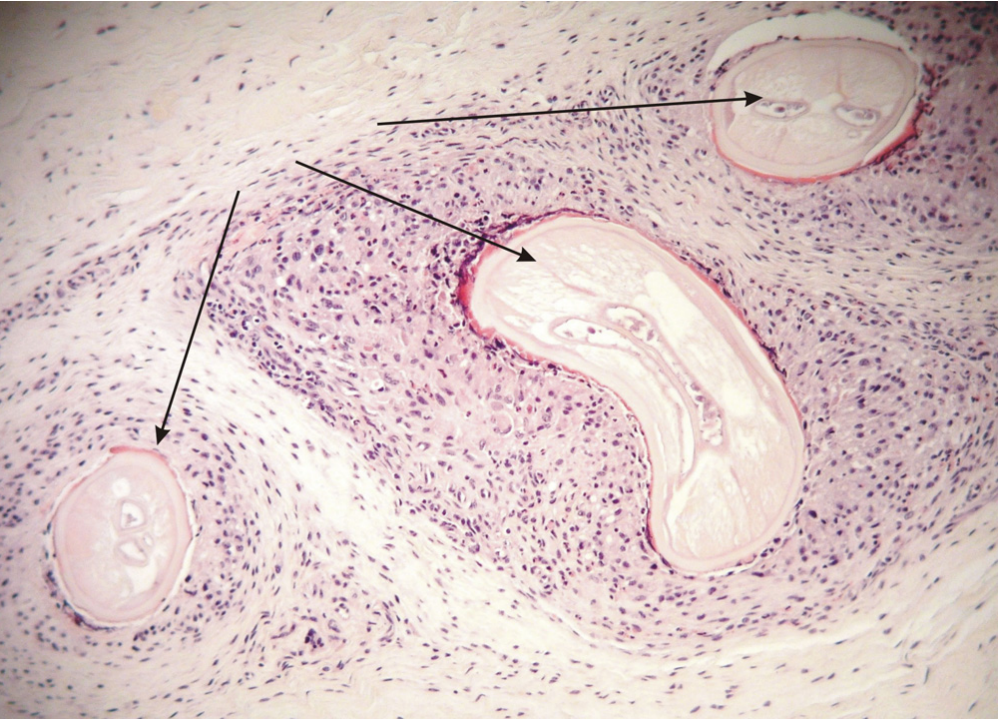
**Histological section of degenerating *Onchocerca *sp. worm (arrows) in connective tissue surrounding knee.** Inflammatory reactions are more intense around dead or degenerating worms.

**Figure 6:**
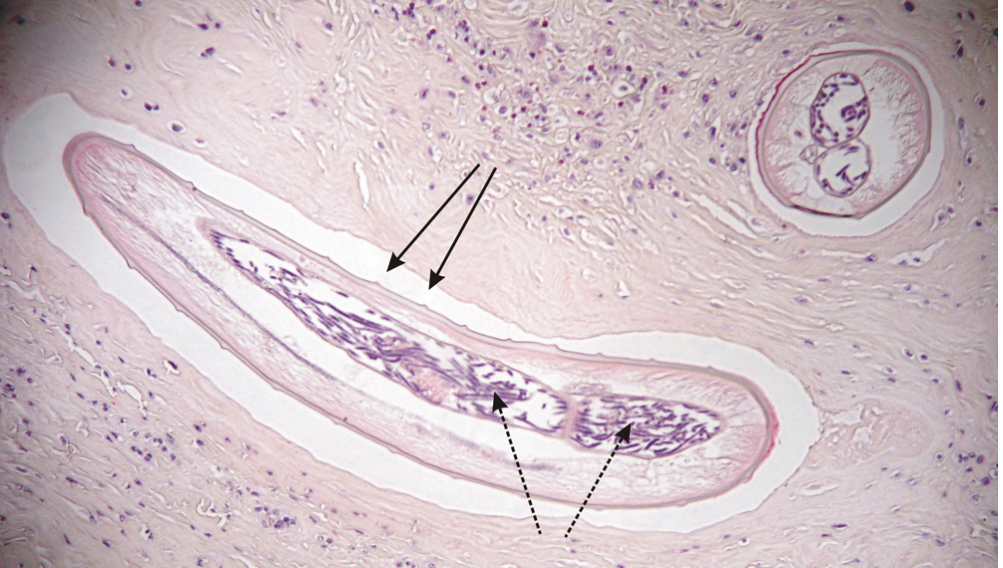
**Histological section of living *Onchocerca *sp. worm (arrows) in connective tissue surrounding knee. **Worm's cuticula has longitudinal ridges and inside the worm numerous microfilariae are seen (spotted arrows). Inflammatory reactions around the living nematode are weak.

The histopathologic examination of the samples of muscle, fascia and connective tissue from legs, flank or brisket revealed mild to intense infiltration with eosinophilic granulocytes, the most intensive lesions were situated perivascularly. Also multifocal nodular lymphoplasmacytic aggregations were seen. In ten of the samples there were also granulomatotic lesions with central necrotic tissue and cell detritus, surrounded by eosinophilic granulocytes, lympho-plasma- and histiocytes and some multinucleated giant cells. Remnants of nematode parasites were seen in three (Fig 5), and mineralised cell detritus in another three samples in the centre of the granulomas. In one sample from near the knee, there were numerous cross sections of living nematodes identified as *Onchocerca *sp. due to their size (70–130 μm in diameter) and because they had a cuticula with longitudinal ridges. Inside some of the cross sections there were numerous mf (Fig. 6). Around the living nematodes only weak or no inflammatory changes were observed. In the liver samples there were granulomatous lesions with necrosis, bleedings and cell detritus, mostly eosinophilic granulocytes, in the centre. Around the necrotic centre, there were lots of eosinophilic granulocytes, various, usually small, numbers of multinucleate giant cells, histio-, lympho- and plasmacytes and mild fibrosis. Near the lesions there were also eosinophilic infiltrations, localized lymphoplasmacytic aggregations, mild fibrosis and cholangial hyperplasia in the periportal areas.

In the lung samples there were localized mild peribronchial lymphoplasmacytic and eosinophilic infiltrations. In one lung sample there were also focal peribronchial eosinophilic inflammatory changes with mild fibrosis.

On the surface of the spleen there were granulomatous lesions with some cross-sections of dead nematodes surrounded by multinucleated giant cells, eosinophilic granulocytes, lympho-, plasma-, histio- and fibrocytes.

## Discussion

Because of the meat inspection data, the finding of Onchocerca sp. microfilariae from skin samples of cattle was expected although the high prevalence (37%) was a surprise. However, in Europe *Onchocerca *spp. infections in cattle are quite abundant; for example in Germany the mf prevalence in one study was 40.4% [25] and in North Wales 28.5% [26].

Although species of *Onchocerca *have generally been considered low-pathogenic and therefore of little veterinary interest [3], according to the current results it is obvious that they inflict pathological changes which lead to condemnation of infected parts of carcasses in bovine meat inspection. The current situation in Finland, according to this study, is not alarming. Pronounced pathological lesions occur in meat inspection infrequently and the majority of lesions are so mild that they often escape the eye of meat inspecting veterinarians. Based on this study it is difficult to evaluate the economical losses onchocercosis may inflict, but it is important to monitor the situation and its development. In histological examination it was seen that the most intense pathological changes were located around dead and degenerating worms, around the living worms, inflammatory reactions were mild or not seen at all. Thus our results are congruent with the prevailing view [2]. Parasitic lesions were seen in organ samples (liver, spleen, lungs). The causative agent could not be identified, but the lesions might have been caused by migrating immature *Onchocerca *worms.

The infection was more abundant in the south which may be caused by the cow densities being higher there than in the north. The studies with *Setaria tundra *revealed that in beginning of a recent outbreak, the infection was more prevalent in the southern reindeer herding area but proceeded towards north [23].

In North Cameroon, where there is a high transmission rate, the prevalence of *Onchocerca *infection declined in old animals, possibly indicating acquired resistance [3]. In this study the age had no significant effect on the mf prevalence indicating that the infection is evenly distributed among old and young animals and that there seems to be no developing immunity during the infection.

The two most abundant *Onchocerca *species infecting cattle in Europe are *O. lienalis *and *O. gutturosa *[26,25,27] which are both distributed worldwide. *Onchocerca gutturosa *lives in the loose connective tissues of the nuchal ligament and other tendons whereas *O. lienalis *lives in the gastrosplenic region of the peritoneal cavity. They both utilize umbilical area as one of their microfilarial predilection site. Microfilariae found in this study closely resemble both *O. gutturosa and O. lienalis *which are difficult to distinguish by morphological criteria [25]. *Onchocerca gutturosa *mf are approximately 270 μm long and 3–5 μm in width whereas *O. lienalis *are 236 μm long and 5–7 μm wide [28]. Both have bent tail, *O. gutturosa *with sharper ankle than *O. lienalis*. The parasitic lesions found in this study were mostly from tendons, ligaments and fasciae of hindlegs which indicates *O. gutturosa*. From one spleen sample, dead nematodes were found; they could be *O. lienalis*. The possibility of the reindeer parasite *O. tarsicola *being the causative agent of the parasitic lesions was rejected because its mf are exceptionally large (400 × 11 μm) [29].

According to this study, umbilical area was considered the cattle mf predilection site, compared to ears.

It is possible that all the *Onchocerca *sp. mf found in cattle in this study belong to one species; (*O. gutturosa *or *O. lienalis*, which are considered synonymous by some authors, although they have not been reported to cause nodules [30]), but there may also be several species infecting cattle in Finland. To investigate this, it would be important to extract the adult worm and examine it both with morphological and molecular biological methods. This could not be done in this study because no living nematodes were found in the lesions. This reasserts the histological findings that inflammatory reactions appear only around the dead or degenerating worms. To our knowledge, there are no previous studies of *Onchocerca *spp. infecting cattle in Fennoscandia to compare our results with.

In this study there was no indication of *Parafilaria *spp. or *Stephanofilaria *spp. nematodes in cattle. Both parasites inflict wounds and bleeding due to the female which penetrates the skin to oviposit eggs or larvae [1]. The sores occur on cattle in summer when the parasite vector flies are active. In this study, the data was mainly collected in the spring and there were no observations on sores. Although summer sore in cattle teats in Finland is evidently not an uncommon phenomenon [8], the aetiology of the disease is still uncertain and requires more investigation.

Because all of the examined blood samples of cattle, horses and sheep were negative, there is no evidence of setariosis or elaeophorosis in domestic animals in Finland. *Onchocerca *sp. mf were not found in horse skin biopsies. However, only 42 samples were collected and examined. This is not enough to eliminate the possibility of onchocercosis in horses in Finland especially as a previous report [16] states that *Onchocerca cervicalis *was fairly common in Finland in 1940's.

*Onchocerca *spp. nematodes are transmitted by black flies (Simuliidae) and biting midges (Ceratopogonidae) [1]. It is obvious that during summer, grazing cattle are exposed to various amounts of harassment by different blood sucking insects. In the past, grazing of dairy cattle was common in Finland, but has become less popular because it is sometimes easier to keep the cows housed all year round. Since 2006, because of the new animal welfare legislation, dairy cows and heifers must have the opportunity to graze or exercise outside at least 60 days during the grazing season from May to September. This is considered to improve the welfare of cattle but also exposes them to the vectors of filarioid parasites. Recent studies with filarioid nematodes indicate that they are perhaps getting more common in the subarctic zone because of climate change (Laaksonen 2008, unpublished). Due to the warming of earth surface and increasing precipitation, the conditions are improving for the vectors of filarioid nematodes and favouring the parasites' transmission and development in the vectors.

## Conclusion

*Onchocerca *sp. infection in cattle is fairly common in Finland, but the amount of pathological changes leading to condemnation of infected parts is low compared to the mf prevalence. Pronounced pathological changes are distinct but mild changes are difficult to distinguish. In the future it would be important to follow the development of the situation by improving the monitoring of changes in meat inspection. Also its pathological significance should be determined. Future challenges are also in recognizing the *Onchocerca *species infecting cattle, its vectors and possible prevention. According to this study, horses and sheep may be free from filarioid nematodes in Finland.

## Competing interests

The authors declare that they have no competing interests.

## Authors' contributions

MS participated in the collection of the samples, did all analyzes for identification of microfilariae from skin biopsies and blood and drafted the manuscript, SL participated in the design and coordination of the study, helped in the collection of the samples and helped to draft the manuscript, MN did all the pathological examinations and wrote parts of the chapters "Material and Methods" and "Results", EP and RA partly coordinated the study and delivered numerous samples for examination, AO helped to draft the manuscript. All authors read and approved the final and revised manuscript.
